# Comprehensive analysis of common mitochondrial DNA variants and colorectal cancer risk

**DOI:** 10.1038/sj.bjc.6604805

**Published:** 2008-12-02

**Authors:** E Webb, P Broderick, I Chandler, S Lubbe, S Penegar, I P M Tomlinson, R S Houlston

**Affiliations:** 1 Section of Cancer Genetics, Institute of Cancer Research, Sutton, Surrey SM2 5NG, UK; 2 Molecular and Population Genetics Laboratory, London Research Institute, Cancer Research UK, London WC2A 3PX, UK

**Keywords:** colorectal cancer, mitochondria, risk

## Abstract

Several lines of evidence implicate mitochondrial dysfunction in the development of cancer. To test the hypothesis that common mtDNA variation influences the risk of colorectal cancer (CRC), we genotyped 132 tagging mtDNA variants in a sample of 2854 CRC cases and 2822 controls. The variants examined capture ∼80% of mtDNA common variation (excluding the hypervariable D-loop). We first tested for single marker associations; the strongest association detected was with A5657G (*P*=0.06). Overall the distribution of association *P-*values was consistent with a null distribution. Next, we classified individuals into the nine common European haplogroups and compared their distribution in cases and controls. This analysis also provided no evidence of an association between mitochondrial variation and CRC risk. In conclusion, our results provide little evidence that mitochondrial genetic background plays a role in modifying an individual's risk of developing CRC.

Approximately 35% of colorectal cancer (CRC) can be ascribed to inherited susceptibility (Lichtenstein *et al*, 2000). Mendelian predisposition syndromes associated with mutations in known genes (*APC*, DNA mismatch repair (MMR) genes, *MYH*, *SMAD4*, *ALK3* and *STK11/LKB1*); however, account for <6% of the overall incidence of the disease (Aaltonen *et al*, 2007). The recent advent of genome-wide association studies has lead to the discovery of several common, low-penetrance susceptibility loci for CRC (Broderick *et al*, 2007; Tomlinson *et al*, 2007, 2008; Tenesa *et al*, 2008), thereby providing incontrovertible evidence for common genetic variation as a basis for CRC susceptibility.

There is increasing evidence that common variation in the mitochondrial DNA (mtDNA) may be functionally relevant to the development of a range of common diseases. Notably, mtDNA polymorphisms have been implicated in a variety of late-onset diseases, including type 2 diabetes (Lowell and Shulman, 2005), Alzheimer's, and Parkinson's disease (Schapira, 1999).

Mitochondria play an essential role in energy metabolism, the generation of reactive oxygen species (ROS) and the regulation of apoptosis (Wallace, 2005), all of which have been implicated in the development of a number of different cancers (Benhar *et al*, 2002). Low levels of ROS regulate cellular signalling and are essential for normal cell proliferation; ROS production is increased in tumour cells causing oxidative stress and DNA damage, which can lead to genetic instability (Burdon, 1995). Thus, ROS are thought to play multiple roles in the initiation, progression, and maintenance of tumours.

Somatic mtDNA mutations can be identified in a wide variety of malignancies, including CRC (Chatterjee *et al*, 2006), although it is unclear whether these are causal or a consequence of the neoplastic process. Given the essential role of mitochondria in ROS generation and regulation of apoptosis, it is however plausible that variant mitochondrial function may directly contribute to an individual's risk of developing cancer. Such an assertion is supported by a recent report implicating polymorphic mtDNA variants in susceptibility to breast cancer (Bai *et al*, 2007).

To date no comprehensive evaluation of the hypothesis that common mtDNA variants influence the risk of developing CRC has been conducted. To address this we have genotyped 132 tagging mtDNA variants, which capture ∼80% of all of the common mitochondrial variation and compared their frequencies in 2854 CRC cases and 2822 controls.

## Materials and methods

### Subjects and samples

A total of 2863 CRC cases (1196 men, 1667 women; mean age at diagnosis 59.3 years; s.d.±8.7) were ascertained through The National Study of Colorectal Cancer Genetics (NSCCG). A total of 2838 healthy individuals were recruited as part of ongoing National Cancer Research Network genetic epidemiological studies, NSCCG (1219), the Genetic Lung Cancer Predisposition Study (GELCAPS) (1999–2004; *n*=911), and the Royal Marsden Hospital Trust/Institute of Cancer Research Family History and DNA Registry (1999–2004; *n*=708). These controls (1136 men, 1702 women; mean age 59.8 years; s.d.±10.8) were the spouses or unrelated friends of patients with malignancies. None had a personal history of malignancy at the time of ascertainment. All cases and controls were British and of European descent, and there were no obvious differences in the demography of cases and controls in terms of place of residence within the United Kingdom. Collection of blood samples and clinico-pathological information from patients and controls was undertaken with informed consent and the ethical review board approval in accordance with the tenets of the Declaration of Helsinki.

### Variant selection and genotyping

DNA was extracted from EDTA venous blood samples using conventional methodologies and quantified using PicoGreen (Invitrogen, Paisley, UK). We excluded the ∼0.8 kb of the hypervariable mtDNA D-loop promoter region/control region from the study, as variation in this region can only realistically be addressed by sequencing because of the high mutation rate associated with this region of the mitochondrial genome. A recent study identified 144 variants with frequency >1% in Europeans and defined a set of 64 single nucleotide polymorphisms (SNPs), which tag all common variants with *r*^2^>0.8 (Saxena *et al*, 2006). On the basis of these data and designability scores for the genotyping platform, we selected 132 tag SNPs, which maximally capture common mtDNA variation.

Genotyping was conducted using Illumina Infinium Bead Arrays according to the manufacturer's protocols. A DNA sample was deemed to have failed if it generated genotypes at fewer than 95% of loci. An SNP was deemed to have failed if fewer than 95% of DNA samples generated a genotype at the locus. To ensure quality of genotyping, a series of duplicate samples were genotyped.

The nucleotide positions presented are taken from the NC_001807 mitochondrial reference sequence in dbSNP. The mapping between this sequence and the revised Cambridge reference sequence for each of the 132 variants tested is detailed in Supplementary Table 1. European mtDNA haplogroups H, I, J, K, T, U, V, W and X were classified according to the published references and the Mitomap database (Torroni *et al*, 1996; Macaulay *et al*, 1999; Herrnstadt *et al*, 2002) (Table 1).

Microsatellite instability in CRCs was determined using the following methodology: 10 *μ*m sections were cut from formalin-fixed paraffin-embedded tumours, lightly stained with toluidine blue, and regions containing at least 60% tumour micro-dissected. Tumour DNA was extracted using the QIAamp DNA Mini kit (Qiagen, Crawley, UK) according to the manufacturer's instructions and genotyped for the mononucleotide microsatellite loci BAT25 and BAT26, which are highly sensitive markers of MSI. Samples showing novel alleles at either BAT26 or BAT25 or both markers were assigned as MSI (corresponding to a high level of instability, MSI-H (Boland *et al*, 1998)).

### Statistical and bioinformatic methods

For several of the SNPs, the rare variant was observed in less than 1% of samples. These variants were excluded from further analysis. We employed the program Tagger (de Bakker *et al*, 2005) to estimate the approximate proportion of common mitochondrial variation defined by the 144 variants described by Saxena *et al* (2006), which was captured by the variants genotyped in our study.

For each individual SNP and haplogroup, comparison of genotype frequencies (or presence/absence of haplogroup frequencies) in cases and controls was initially undertaken using a *χ*^2^-test with one degree of freedom and unadjusted odds ratios (ORs) were calculated. We used logistic regression to calculate ORs adjusted for age and gender, and their associated 95% confidence intervals. For each SNP, a one-degree of freedom likelihood ratio test comparing the model including covariates age and gender with the model including covariates age, gender and SNP genotype was performed.

Correction for multiple testing in association studies using a simple Bonferroni correction may be conservative due to the assumption of independence between tests. We therefore adopted an empirical simulation approach based on 10 000 permutations, thus allowing for correlations between mtDNA variants. At each iteration case and control labels were permuted at random and the maximum likelihood ratio test statistic calculated. The significance level for each SNP was estimated as the proportion of permutation samples for which this maximum was larger than the observed value.

We assessed the possibility of interactive effects between each pair of SNPs that displayed some evidence of association (*P*<0.1) by computing the likelihood ratio test statistic for the saturated model against the main effects model. We also assessed the possibility that the effect of each SNP on CRC risk was modified by age by computing the likelihood ratio test statistic for the model with a genotype-age interaction against the model with genotype and age terms only.

A number of additional covariates were available for the CRC cases, including family history of CRC (at least one first-degree relative with CRC), site of tumour (colon/rectum) and MSI status. For each SNP and haplogroup, we assessed the association with CRC risk restricted to case subgroups defined by these covariates. For each subgroup, logistic regression was used to estimate ORs adjusted for age and gender and likelihood ratio test statistics were calculated. All statistical analyses were undertaken in R v.2.4.

## Results

Out of the 5701 DNA samples submitted for genotyping, 5676 samples were successfully processed. Genotyping failed in 25 individuals, leaving genotype data for 2854 cases and 2822 controls.

Of the 132 variants for which genotyping were attempted, 125 were satisfactorily genotyped (94.7%), with mean SNP call rates of 99.9 and 99.8% in cases and controls, respectively. Of these 125 SNPs, eight were monomorphic and an additional 54 had the minor variant observed in less than 1% of samples and were excluded from further analysis, leaving 63 polymorphic variants. Only one SNP (A15925G) was triallelic in samples analysed with one heterozygote observed among cases. This genotype was treated as missing for the analysis. Genotypes from duplicate samples displayed 100% concordance.

One variant (G10590A), polymorphic in our samples was not observed by (Saxena *et al*, 2006), and nine variants observed to be polymorphic in their study were either monomorphic or had very low frequency in our samples (Supplementary Table 2). Given these caveats, our data indicated that 79.3% of polymorphic variants were captured with *r*^2^>0.8, whereas 92.2% of variants with MAF>5% were captured with *r*^2^>0.8.

Four SNPs showed nominal levels of association with CRC risk (*P*<0.1; Table 2). The most strongly associated was A5657G, with a *P*-value of 0.06; non-significant after adjustment for multiple testing by permutation. All nine common European haplogroups (H, I, J, K, T, U, V, W and X) were observed in both cases and controls. Haplogroup J was slightly over-represented in cases, whereas haplogroup K was slightly under-represented, although these observations were statistically non-significant (Table 3). Adjustment for age and gender did not impact on the findings.

Interactions between the four SNPs that showed an association with CRC risk at the 10% level of significance were examined by fitting full logistic regression models for each pair, generating six models, and comparing with the main effects model for each pair. Owing to small MAFs, it was only possible to evaluate the interaction for three of the pairs. For each of these there was no significant evidence of interactive effects. Furthermore, there was no evidence of any differential effect of genotype by either age or gender.

For all 2854 genotyped cases, information was available on site of CRC (1743 colonic, 1111 rectal tumours) and family history (398 individuals with at least one first-degree relative affected by CRC, 2456 with no recorded family history), and 1222 of the cases had been evaluated for MSI status (151 MSI, 1071 MSS cases). Subgroup analysis by site indicated stronger evidence of association between mtDNA variants and colon cancer, with five variants showing significant association (*P*<0.05) whereas there was no evidence for an association between any variant and rectal cancer (*P*>0.1 for all variants). The variant A5657G was most strongly associated with the risk of colonic tumour (*P*=0.02), albeit non-significant after adjustment for multiple testing. Stratification by MSI status showed that three variants were associated with risk of CRC for MSI cases, with the strongest association for T4562C (*P*=4.6 × 10^−3^), non-significant after adjustment for multiple testing. There was no evidence for association between any SNP and CRC in MSS cases (*P*>0.05 for all variants). Stratification by family history status did not alter the overall findings.

## Discussion

It is entirely plausible that genetic variation in mitochondrial genome might influence cancer risk given the increasing evidence implicating hypoxia in the development of cancer and the pivotal role of mitochondrial function in cellular energy metabolism.

Previous studies have tested small numbers of mtDNA variants for an association with a variety of traits, typically focusing on the nine canonical haplogroups, with limited tagging coverage generally capturing <40% of common variation (*r*^2^>0.8). To generate a more comprehensive analysis of the relationship between mitochondrial variation and CRC risk we have analysed variants that capture 79% of all polymorphic variants (MAF>1%) and 92% of variants with MAF>5% (*r*^2^>0.8). A further strength of our study is that our analysis has been based on a large case–control series. We genotyped 132 mtDNA variants and analysed data from the 63 variants with frequencies >1%. Under the assumption that the 63 tests were independent, our study therefore had 70% power to detect a variant with a frequency of 5% conferring a 1.5-fold increase in risk of CRC. Moreover, for variants with MAFs of 10% or greater, our study had >80% power to identify variants conferring a 1.3-fold increase in risk.

Despite our study being a well-powered evaluation capturing the majority of common variation in mtDNA, our findings do not support the hypothesis that common mtDNA variants play a significant role in inherited CRC. Specifically, results from our association tests of all common mtDNA variants and the risk of CRC show that there is no single common coding-region mtDNA variant or haplogroup that strongly influences risk of developing CRC. It is however, entirely possible that any genetic variation in mitochondria influencing CRC risk may be in the form of low frequency variants, although we have no evidence from our data that this is the case. Alternatively the impact of variants may be restricted to a subset of CRC, as there are differences in the biological basis of CRC according to site.

Observations based on *post hoc* analyses are inherently prone to generating spurious associations. Accepting such caveats it is, however, noteworthy that we found a stronger relationship between A5657G and colonic rather than rectal disease. There was also evidence for an association between risk of MSI CRC and T4562C. Tumour hypoxia has been reported to cause a functional loss of DNA mismatch repair system as a result of downregulation of MMR genes, principally involving *MLH1* (Mihaylova *et al*, 2003; Bindra *et al*, 2007; Nakamura *et al*, 2008) thereby in keeping with the observation. Although attractive, such a postulate requires validation in additional independent datasets. As A5657G is non-coding and T4562C is a synonymous change, any effect is likely to be indirect, which is possibly mediated through an untyped SNP.

A limitation of our study is that it does not address the role of mtDNA heteroplasmy in CRC. Typically, blood DNA exhibits much less heteroplasmy than non-dividing tissues. Indeed in the 5676 DNA samples genotyped, only one heterozygote call was observed although it is possible that this is because of analytical limitations of the platform employed. However, as the known rare mitochondrial diseases exhibit pronounced heteroplasmy, it is unlikely that mtDNA heteroplasmy for such variants will have significantly influenced our findings.

In conclusion, our results provide no support that common mtDNA variation plays a role in inherited predisposition to CRC. It is however, possible that mitochondria may be involved in gene–gene and gene–environment interactions that may affect disease risk. To address such hypotheses requires studies based on very large sample sizes that incorporate data on non-genetic covariates.

## Figures and Tables

**Table 1:** Classification of haplogroups

**Haplogroup**	**G1721A**	**T4217C**	**G4581A**	**T10035C**	**G10399A**	**A12309G**	**T14471C**	**T14767C**
H	—	—	—	—	A	—	—	C
I	A	—	—	C	G	—	—	—
J	—	C	—	—	G	—	—	—
K	—	—	—	—	G	G	—	—
T	—	C	—	—	A	A	—	—
U	—	—	—	—	A	G	—	—
V	—	—	A	—	A	—	—	C
W	—	—	—	—	A	—	—	—
X	A	—	—	—	A	—	C	—

**Table 2:** Relationship between CRC risk and the sixty-three common mtDNA variants

	**MAF**		**Adjusted results^a^**		**Unadjusted results**	
**SNP**	**Case**	**Control**	***P*-value**	**OR (95% CI)**	***P*-value**	**OR (95% CI)**
G752A	0.018	0.018	0.99	1.00 (0.68, 1.47)	0.95	0.99 (0.67, 1.46)
G1440A	0.033	0.027	0.18	1.24 (0.91, 1.68)	0.19	1.23 (0.90, 1.67)
G1721A	0.058	0.054	0.54	1.07 (0.86, 1.35)	0.52	1.08 (0.86, 1.35)
T2160C	0.014	0.012	0.61	1.13 (0.71, 1.78)	0.60	1.13 (0.72, 1.79)
G2708A	0.434	0.438	0.75	0.98 (0.88, 1.09)	0.80	0.99 (0.89, 1.10)
G3012A	0.256	0.247	0.41	1.05 (0.93, 1.19)	0.41	1.05 (0.93, 1.19)
T3198C	0.102	0.089	0.10	1.16 (0.97, 1.39)	0.12	1.15 (0.96, 1.38)
A3481G	0.080	0.094	0.09	0.85 (1.02, 0.71)	0.07	0.85 (0.70, 1.02)
A3721G	0.011	0.012	0.55	0.86 (1.40, 0.53)	0.59	0.87 (0.54, 1.42)
G3916A	0.026	0.026	0.88	0.98 (0.70, 1.36)	0.94	0.99 (0.71, 1.37)
C3993T	0.025	0.023	0.65	1.08 (1.53, 0.77)	0.72	1.07 (0.76, 1.50)
A4025G	0.019	0.020	0.75	0.94 (1.37, 0.64)	0.65	0.92 (0.63, 1.34)
T4217C	0.213	0.203	0.32	1.07 (0.94, 1.22)	0.39	1.06 (0.93, 1.20)
T4337C	0.025	0.025	0.99	1.00 (0.72, 1.40)	0.99	1.00 (0.72, 1.40)
T4562C	0.012	0.012	0.99	1.00 (0.62, 1.61)	0.96	0.99 (0.61, 1.59)
G4581A	0.037	0.039	0.64	0.94 (0.71, 1.23)	0.72	0.95 (0.72, 1.25)
G4770A	0.030	0.025	0.20	1.23 (0.90, 1.69)	0.22	1.22 (0.89, 1.67)
A4918G	0.100	0.105	0.58	0.95 (1.13, 0.80)	0.51	0.94 (0.79, 1.12)
T5005C	0.020	0.023	0.60	0.91 (0.63, 1.30)	0.54	0.89 (0.62, 1.28)
A5391G	0.011	0.011	0.80	0.94 (1.54, 0.57)	0.87	0.96 (0.58, 1.57)
G5461A	0.055	0.047	0.21	1.16 (0.92, 1.48)	0.19	1.17 (0.92, 1.48)
T5496C	0.014	0.015	0.67	0.91 (0.59, 1.41)	0.62	0.90 (0.58, 1.39)
A5657G	0.016	0.010	0.06	1.58 (2.56, 0.98)	0.06	1.57 (0.97, 2.52)
C6046T	0.011	0.011	0.80	0.94 (1.54, 0.57)	0.86	0.96 (0.58, 1.57)
T6153C	0.012	0.012	0.90	0.97 (0.60, 1.58)	0.97	0.99 (0.61, 1.61)
T6222C	0.016	0.013	0.27	1.28 (0.82, 1.98)	0.29	1.27 (0.82, 1.97)
G6261A	0.018	0.019	0.83	0.96 (0.65, 1.41)	0.80	0.95 (0.65, 1.40)
C6372T	0.015	0.011	0.22	1.34 (2.12, 0.84)	0.22	1.33 (0.84, 2.11)
G6735A	0.012	0.010	0.45	1.22 (0.73, 2.01)	0.47	1.20 (0.73, 1.99)
A7769G	0.046	0.038	0.11	1.24 (1.61, 0.95)	0.13	1.22 (0.94, 1.58)
G8270A	0.034	0.032	0.61	1.08 (0.81, 1.45)	0.66	1.07 (0.80, 1.43)
G8698A	0.098	0.103	0.61	0.96 (0.80, 1.14)	0.53	0.95 (0.80, 1.12)
A9668G	0.014	0.012	0.48	1.18 (1.86, 0.75)	0.52	1.16 (0.74, 1.83)
T9699C	0.084	0.096	0.12	0.86 (0.72, 1.04)	0.10	0.86 (0.72, 1.03)
T9900C	0.014	0.017	0.41	0.84 (0.55, 1.28)	0.42	0.84 (0.55, 1.28)
T10035C	0.037	0.033	0.37	1.14 (0.86, 1.51)	0.32	1.15 (0.87, 1.53)
A10045G	0.011	0.011	0.79	1.07 (1.78, 0.65)	0.94	1.02 (0.62, 1.69)
T10239C	0.036	0.035	0.99	1.00 (0.76, 1.33)	0.90	1.02 (0.77, 1.35)
G10399A	0.213	0.211	0.87	1.01 (1.15, 0.89)	0.85	1.01 (0.89, 1.15)
T10464C	0.105	0.109	0.69	0.97 (0.82, 1.14)	0.59	0.95 (0.81, 1.13)
A10551G	0.079	0.093	0.08	0.85 (1.02, 0.70)	0.07	0.84 (0.70, 1.01)
G10590A	0.014	0.015	0.62	0.90 (0.58, 1.39)	0.62	0.90 (0.58, 1.38)
T10916C	0.012	0.011	0.82	1.06 (0.65, 1.72)	0.84	1.05 (0.65, 1.71)
G11378A	0.020	0.020	0.87	0.97 (0.67, 1.41)	0.88	0.97 (0.67, 1.41)
A11468G	0.222	0.228	0.66	0.97 (1.10, 0.86)	0.59	0.97 (0.85, 1.09)
T11486C	0.021	0.019	0.61	1.10 (0.76, 1.60)	0.61	1.10 (0.76, 1.60)
A11813G	0.082	0.084	0.90	0.99 (1.19, 0.82)	0.79	0.97 (0.81, 1.18)
G11915A	0.018	0.021	0.56	0.89 (0.61, 1.31)	0.52	0.88 (0.61, 1.29)
A12309G	0.223	0.229	0.70	0.98 (1.11, 0.86)	0.64	0.97 (0.86, 1.10)
G12373A	0.224	0.229	0.69	0.97 (0.86, 1.10)	0.62	0.97 (0.86, 1.10)
T12415C	0.019	0.020	0.72	0.93 (0.64, 1.36)	0.80	0.95 (0.66, 1.39)
T12706C	0.074	0.072	0.91	0.99 (0.81, 1.21)	0.81	1.02 (0.84, 1.25)
A13781G	0.035	0.034	0.93	1.01 (1.35, 0.76)	0.83	1.03 (0.77, 1.37)
A14234G	0.082	0.085	0.83	0.98 (1.18, 0.81)	0.72	0.97 (0.80, 1.17)
T14471C	0.019	0.013	0.09	1.43 (0.94, 2.17)	0.08	1.44 (0.95, 2.18)
T14767C	0.492	0.498	0.57	0.97 (0.87, 1.08)	0.64	0.98 (0.88, 1.08)
T14799C	0.162	0.164	0.94	0.99 (0.86, 1.15)	0.85	0.99 (0.86, 1.14)
G15044A	0.042	0.045	0.57	0.93 (0.72, 1.20)	0.63	0.94 (0.73, 1.21)
A15219G	0.041	0.041	0.99	1.00 (1.30, 0.77)	0.98	1.00 (0.77, 1.30)
C15834T	0.021	0.021	0.99	1.00 (1.44, 0.70)	0.98	1.01 (0.70, 1.45)
C15905T	0.037	0.039	0.57	0.92 (1.22, 0.70)	0.66	0.94 (0.72, 1.24)
A15925G	0.061	0.062	0.80	0.97 (1.21, 0.78)	0.90	0.99 (0.79, 1.23)
G15929A	0.100	0.104	0.64	0.96 (0.81, 1.14)	0.56	0.95 (0.80, 1.13)

CI=confidence interval; MAF=minor allele frequency; OR=odds ratio.

a Adjusted for age and gender.

**Table 3:** Risk of CRC associated with the nine common European haplogroups

	**Frequency (%)**		**Adjusted results^a^**		**Unadjusted results**	
**Haplogroup**	**Case**	**Control**	***P*-value**	**OR (95% CI)**	***P*-value**	**OR (95% CI)**
H	1258 (44.1)	1261 (44.7)	0.59	0.99 (0.94–1.04)	0.65	0.99 (0.94–1.04)
I	98 (3.4)	88 (3.1)	0.57	1.04 (0.90–1.21)	0.50	1.05 (0.91–1.22)
J	300 (10.5)	263 (9.3)	0.12	1.07 (0.98–1.17)	0.13	1.07 (0.98–1.17)
K	184 (6.4)	213 (7.5)	0.10	0.92 (0.83–1.02)	0.10	0.92 (0.83–1.02)
T	303 (10.6)	306 (10.8)	0.91	1.00 (0.91–1.08)	0.78	0.99 (0.91–1.07)
U	452 (15.8)	430 (15.2)	0.47	1.03 (0.96–1.10)	0.53	1.02 (0.95–1.10)
V	104 (3.6)	108 (3.8)	0.64	0.97 (0.84–1.11)	0.72	0.97 (0.85–1.12)
W	67 (2.3)	76 (2.7)	0.33	0.92 (0.78–1.09)	0.41	0.93 (0.79–1.10)
X	42 (1.5)	31 (1.1)	0.21	1.16 (0.92–1.47)	0.21	1.16 (0.92–1.46)
Undefined	46 (1.6)	46 (1.6)	1.00	1.00 (0.81–1.23)	0.96	0.99 (0.81–1.22)

CI=confidence interval; OR=odds ratio.

a Adjusted for age and gender.

## References

[bib1] Aaltonen L, Johns L, Jarvinen H, Mecklin JP, Houlston R (2007) Explaining the familial colorectal cancer risk associated with mismatch repair (MMR)-deficient and MMR-stable tumors. Clin Cancer Res 13: 356–361 17200375 10.1158/1078-0432.CCR-06-1256

[bib2] Bai RK, Leal SM, Covarrubias D, Liu A, Wong LJ (2007) Mitochondrial genetic background modifies breast cancer risk. Cancer Res 67: 4687–4694 17510395 10.1158/0008-5472.CAN-06-3554

[bib3] Benhar M, Engelberg D, Levitzki A (2002) ROS, stress-activated kinases and stress signaling in cancer. EMBO Rep 3: 420–425 11991946 10.1093/embo-reports/kvf094 PMC1084107

[bib4] Bindra RS, Crosby ME, Glazer PM (2007) Regulation of DNA repair in hypoxic cancer cells. Cancer Metastasis Rev 26: 249–260 17415527 10.1007/s10555-007-9061-3

[bib5] Boland CR, Thibodeau SN, Hamilton SR, Sidransky D, Eshleman JR, Burt RW, Meltzer SJ, Rodriguez-Bigas MA, Fodde R, Ranzani GN, Srivastava S (1998) A National Cancer Institute Workshop on Microsatellite Instability for cancer detection and familial predisposition: development of international criteria for the determination of microsatellite instability in colorectal cancer. Cancer Res 58: 5248–5257 9823339

[bib6] Broderick P, Carvajal-Carmona L, Pittman AM, Webb E, Howarth K, Rowan A, Lubbe S, Spain S, Sullivan K, Fielding S, Jaeger E, Vijayakrishnan J, Kemp Z, Gorman M, Chandler I, Papaemmanuil E, Penegar S, Wood W, Sellick G, Qureshi M, Teixeira A, Domingo E, Barclay E, Martin L, Sieber O, Kerr D, Gray R, Peto J, Cazier JB, Tomlinson I, Houlston RS (2007) A genome-wide association study shows that common alleles of SMAD7 influence colorectal cancer risk. Nat Genet 39: 1315–1317 17934461 10.1038/ng.2007.18

[bib7] Burdon RH (1995) Superoxide and hydrogen peroxide in relation to mammalian cell proliferation. Free Radic Biol Med 18: 775–794 7750801 10.1016/0891-5849(94)00198-s

[bib8] Chatterjee A, Mambo E, Sidransky D (2006) Mitochondrial DNA mutations in human cancer. Oncogene 25: 4663–4674 16892080 10.1038/sj.onc.1209604

[bib9] de Bakker PI, Yelensky R, Pe’er I, Gabriel SB, Daly MJ, Altshuler D (2005) Efficiency and power in genetic association studies. Nat Genet 37: 1217–1223 16244653 10.1038/ng1669

[bib10] Herrnstadt C, Elson JL, Fahy E, Preston G, Turnbull DM, Anderson C, Ghosh SS, Olefsky JM, Beal MF, Davis RE, Howell N (2002) Reduced-median-network analysis of complete mitochondrial DNA coding-region sequences for the major African, Asian, and European haplogroups. Am J Hum Genet 70: 1152–1171 11938495 10.1086/339933 PMC447592

[bib11] Lichtenstein P, Holm NV, Verkasalo PK, Iliadou A, Kaprio J, Koskenvuo M, Pukkala E, Skytthe A, Hemminki K (2000) Environmental and heritable factors in the causation of cancer--analyses of cohorts of twins from Sweden, Denmark, and Finland. N Engl J Med 343: 78–85 10891514 10.1056/NEJM200007133430201

[bib12] Lowell BB, Shulman GI (2005) Mitochondrial dysfunction and type 2 diabetes. Science 307: 384–387 15662004 10.1126/science.1104343

[bib13] Macaulay V, Richards M, Hickey E, Vega E, Cruciani F, Guida V, Scozzari R, Bonne-Tamir B, Sykes B, Torroni A (1999) The emerging tree of West Eurasian mtDNAs: a synthesis of control-region sequences and RFLPs. Am J Hum Genet 64: 232–249 9915963 10.1086/302204 PMC1377722

[bib14] Mihaylova VT, Bindra RS, Yuan J, Campisi D, Narayanan L, Jensen R, Giordano F, Johnson RS, Rockwell S, Glazer PM (2003) Decreased expression of the DNA mismatch repair gene Mlh1 under hypoxic stress in mammalian cells. Mol Cell Biol 23: 3265–3273 12697826 10.1128/MCB.23.9.3265-3273.2003 PMC153206

[bib15] Nakamura H, Tanimoto K, Hiyama K, Yunokawa M, Kawamoto T, Kato Y, Yoshiga K, Poellinger L, Hiyama E, Nishiyama M (2008) Human mismatch repair gene, MLH1, is transcriptionally repressed by the hypoxia-inducible transcription factors, DEC1 and DEC2. Oncogene 27(30): 4200–4209 18345027 10.1038/onc.2008.58

[bib16] Saxena R, de Bakker PI, Singer K, Mootha V, Burtt N, Hirschhorn JN, Gaudet D, Isomaa B, Daly MJ, Groop L, Ardlie KG, Altshuler D (2006) Comprehensive association testing of common mitochondrial DNA variation in metabolic disease. Am J Hum Genet 79: 54–61 16773565 10.1086/504926 PMC1474138

[bib17] Schapira AH (1999) Mitochondrial involvement in Parkinson's disease, Huntington's disease, hereditary spastic paraplegia and Friedreich's ataxia. Biochim Biophys Acta 1410: 159–170 10076024 10.1016/s0005-2728(98)00164-9

[bib18] Tenesa A, Farrington SM, Prendergast JG, Porteous ME, Walker M, Haq N, Barnetson RA, Theodoratou E, Cetnarskyj R, Cartwright N, Semple C, Clark AJ, Reid FJ, Smith LA, Kavoussanakis K, Koessler T, Pharoah PD, Buch S, Schafmayer C, Tepel J, Schreiber S, Volzke H, Schmidt CO, Hampe J, Chang-Claude J, Hoffmeister M, Brenner H, Wilkening S, Canzian F, Capella G, Moreno V, Deary IJ, Starr JM, Tomlinson IP, Kemp Z, Carvajal-Carmona L, Webb E, Broderick P, Vijayakrishnan J, Houlston RS, Rennert G, Ballinger D, Rozek L, Gruber SB, Matsuda K, Kidokoro T, Nakamura Y, Zanke BW, Greenwood CM, Rangrej J, Kustra R, Montpetit A, Hudson TJ, Gallinger S, Campbell H, Dunlop MG (2008) Genome-wide association scan identifies a colorectal cancer susceptibility locus on 11q23 and replicates risk loci at 8q24 and 18q21. Nat Genet 40(5): 631–637 18372901 10.1038/ng.133 PMC2778004

[bib19] Tomlinson I, Webb E, Carvajal-Carmona L, Broderick P, Kemp Z, Spain S, Penegar S, Chandler I, Gorman M, Wood W, Barclay E, Lubbe S, Martin L, Sellick G, Jaeger E, Hubner R, Wild R, Rowan A, Fielding S, Howarth K, Silver A, Atkin W, Muir K, Logan R, Kerr D, Johnstone E, Sieber O, Gray R, Thomas H, Peto J, Cazier JB, Houlston R (2007) A genome-wide association scan of tag SNPs identifies a susceptibility variant for colorectal cancer at 8q24.21. Nat Genet 39: 984–988 17618284 10.1038/ng2085

[bib20] Tomlinson IP, Webb E, Carvajal-Carmona L, Broderick P, Howarth K, Pittman AM, Spain S, Lubbe S, Walther A, Sullivan K, Jaeger E, Fielding S, Rowan A, Vijayakrishnan J, Domingo E, Chandler I, Kemp Z, Qureshi M, Farrington SM, Tenesa A, Prendergast JG, Barnetson RA, Penegar S, Barclay E, Wood W, Martin L, Gorman M, Thomas H, Peto J, Bishop DT, Gray R, Maher ER, Lucassen A, Kerr D, Evans DG, Schafmayer C, Buch S, Volzke H, Hampe J, Schreiber S, John U, Koessler T, Pharoah P, van Wezel T, Morreau H, Wijnen JT, Hopper JL, Southey MC, Giles GG, Severi G, Castellvi-Bel S, Ruiz-Ponte C, Carracedo A, Castells A, Forsti A, Hemminki K, Vodicka P, Naccarati A, Lipton L, Ho JW, Cheng KK, Sham PC, Luk J, Agundez JA, Ladero JM, de la Hoya M, Caldes T, Niittymaki I, Tuupanene S, Karhu A, Aaltonen L, Cazier JB, Campbell H, Dunlop MG, Houlston RS (2008) A genome-wide association study identifies colorectal cancer susceptibility loci on chromosomes 10p14 and 8q23.3. Nat Genet 40(5): 623–630 18372905 10.1038/ng.111

[bib21] Torroni A, Huoponen K, Francalacci P, Petrozzi M, Morelli L, Scozzari R, Obinu D, Savontaus ML, Wallace DC (1996) Classification of European mtDNAs from an analysis of three European populations. Genetics 144: 1835–1850 8978068 10.1093/genetics/144.4.1835 PMC1207732

[bib22] Wallace DC (2005) A mitochondrial paradigm of metabolic and degenerative diseases, aging, and cancer: a dawn for evolutionary medicine. Annu Rev Genet 39: 359–407 16285865 10.1146/annurev.genet.39.110304.095751 PMC2821041

