# Supplementary material for: Comprehensive analysis of common mitochondrial DNA variants and colorectal cancer risk
Source: Br J Cancer. 2008 Dec 2;99(12):2088–93. doi: 10.1038/sj.bjc.6604805 (PMC2607223; doi:10.1038/sj.bjc.6604805)
Supplement: Supplementary Table 1 [file 6604805x1.doc]

**Supplementary Table 1.** Common variants identified by Saxena et al.^1^ observed in fewer than 1% of mtDNAs in our study

| **SNP** | **MAF in our study^2^** | **MAF reported by Saxena et al.** |
| --- | --- | --- |
| A3249G  T4640C  T4929C  C5264T  G8617T  A8870G  A9094G  C12670T  T13966C | 0.003  0.002  Rare variant not observed  0.002  0.009  0.001  0.009  Rare variant not observed  0.004 | 0.02  0.02  0.02  0.01  0.01  0.02  0.01  0.02  0.02 |

^1^ Saxena, R., et al., *Comprehensive association testing of common mitochondrial DNA variation in metabolic disease.* Am J Hum Genet, 2006. **79**: p. 54-61.

^2^ in controls
