# Supplementary material for: Comprehensive analysis of common mitochondrial DNA variants and colorectal cancer risk
Source: Br J Cancer. 2008 Dec 2;99(12):2088–93. doi: 10.1038/sj.bjc.6604805 (PMC2607223; doi:10.1038/sj.bjc.6604805)
Supplement: Supplementary Table 2 [file 6604805x2.doc]

**Supplementary Table 2**. Mapping between the NC_001807 mitochondrial reference sequence in dbSNP used in this study and the revised Cambridge reference sequence.

| **Variant name** | **Position in dbSNP reference sequence NC_001807** | **Position in revised Cambridge reference sequence** |
| --- | --- | --- |
| G752A  A829G  G1020A  C1050T  G1440A  G1721A  A1738G  T2160C  C2354T  C2485T  G2708A  G2760A  C2791T  T2887C  G3012A  T3198C  A3349G  A3481G  A3548G  C3595T  G3667A  A3721G  G3916A  G3919A  C3971T  C3993T  A4025G  A4105G  T4217C  T4337C  T4562C  G4581A  T4640C  G4770A  G4821A  A4825G  C4884T  A4918G  T4929C  T5005C  C5264T  A5391G  T5443C  G5461A  T5496C  A5657G  G5774A  A5952G  C6046T  T6072C  T6153C  T6222C  G6261A  T6366C  C6372T  T6720C  G6735A  A6753G  T6777C  A7056G  A7147G  C7257T  C7275T  G7522A  A7769G  G8270A  T8278C  G8617T  C8656T  G8698A  A8870G  A9073G  T9091C  A9094G  A9222G  G9378A  C9541T  A9668G  T9699C  T9900C  T9951C  T10035C  A10045G  T10239C  T10322C  G10399A  T10464C  A10551G  G10587A  G10590A  G10689A  C10874T  T10916C  A11252G  G11378A  A11468G  T11486C  C11723T  A11813G  T11900C  G11915A  A12309G  G12373A  T12415C  G12631A  C12670T  T12706C  G12851A  C13651T  A13781G  T13790C  T13966C  G14017A  T14179C  C14213T  A14234G  T14471C  G14581A  T14767C  T14784C  T14799C  G15044A  G15111A  A15219G  A15245G  C15536T  T15671C  C15834T  C15905T  A15925G  G15929A  G15931A | 752  829  1020  1050  1440  1721  1738  2160  2354  2485  2708  2760  2791  2887  3012  3198  3349  3481  3548  3595  3667  3721  3916  3919  3971  3993  4025  4105  4217  4337  4562  4581  4640  4770  4821  4825  4884  4918  4929  5005  5264  5391  5443  5461  5496  5657  5774  5952  6046  6072  6153  6222  6261  6366  6372  6720  6735  6753  6777  7056  7147  7257  7275  7522  7769  8270  8278  8617  8656  8698  8870  9073  9091  9094  9222  9378  9541  9668  9699  9900  9951  10035  10045  10239  10322  10399  10464  10551  10587  10590  10689  10874  10916  11252  11378  11468  11486  11723  11813  11900  11915  12309  12373  12415  12631  12670  12706  12851  13651  13781  13790  13966  14017  14179  14213  14234  14471  14581  14767  14784  14799  15044  15111  15219  15245  15536  15671  15834  15905  15925  15929  15931 | 750  827  1018  1048  1438  1719  1736  2158  2352  2483  2706  2758  2789  2885  3010  3197  3348  3480  3547  3594  3666  3720  3915  3918  3970  3992  4024  4104  4216  4336  4561  4580  4639  4769  4820  4824  4883  4917  4928  5004  5263  5390  5442  5460  5495  5656  5773  5951  6045  6071  6152  6221  6260  6365  6371  6719  6734  6752  6776  7055  7146  7256  7274  7521  7768  8269  8277  8616  8655  8697  8869  9072  9090  9093  9221  9377  9540  9667  9698  9899  9950  10034  10044  10238  10321  10398  10463  10550  10586  10589  10688  10873  10915  11251  11377  11467  11485  11722  11812  11899  11914  12308  12372  12414  12630  12669  12705  12850  13650  13780  13789  13965  14016  14178  14212  14233  14470  14580  14766  14783  14798  15043  15110  15218  15244  15535  15670  15833  15904  15924  15928  15930 |
